# *Arabidopsis* Voltage-Dependent Anion Channels (VDACs): Overlapping and Specific Functions in Mitochondria

**DOI:** 10.3390/cells9041023

**Published:** 2020-04-21

**Authors:** Mickaële Hemono, Élodie Ubrig, Kevin Azeredo, Thalia Salinas-Giegé, Laurence Drouard, Anne-Marie Duchêne

**Affiliations:** Institut de Biologie Moléculaire des Plantes, UPR 2357 du CNRS, Université de Strasbourg, 12 rue du Général Zimmer, 67084 Strasbourg Cedex, France; mickaele.hemono@etu.unistra.fr (M.H.); Elodie.Ubrig@ibmp-cnrs.unistra.fr (É.U.); kevin.azeredo@etu.unistra.fr (K.A.); thalia.salinas@ibmp-cnrs.unistra.fr (T.S.-G.); Laurence.Drouard@ibmp-cnrs.unistra.fr (L.D.)

**Keywords:** outer membrane, tRNA import, cold stress, heat stress, oxidative stress, phosphate, salt, proteome, oxidative phosphorylation, OXPHOS, oxygen uptake

## Abstract

Voltage-dependent anion channels (VDACs) are essential components of the mitochondrial outer membrane. VDACs are involved in the exchange of numerous ions and molecules, from ATP to larger molecules such as tRNAs, and are supposed to adjust exchanges in response to cell signals and stresses. Four major VDACs have been identified in *Arabidopsis thaliana*. The goal of this study was to explore the specific functions of these proteins, in particular, in tRNA import into mitochondria and stress response. The main results were: (i) VDACs appeared to differentially interact with tRNAs, and VDAC4 could be the major tRNA channel on the outer membrane, (ii) a VDAC3 mRNA isoform was found induced by different stresses, suggesting that VDAC3 might be specifically involved in early steps of stress response and (iii) an analysis of *vdac3* and *vdac1* mutant lines showed that VDAC3 and VDAC1 shared some, but not all functions. In conclusion, this work brings new knowledge on VDACs, which do not appear as interchangeable pores of the outer membrane and each VDAC has its own specificity.

## 1. Introduction

The voltage-dependent anion channels (VDACs) are the major protein components of the mitochondrial outer membrane (MOM). VDACs are involved in the exchange of numerous compounds between the cytosol and the inter membrane space. These compounds are ions such as Ca^2+^, metabolites such as succinate or ATP, and also larger molecules such as tRNA or DNA [1,2,3]. VDACs are pore-forming proteins organized in β-barrels of 19 β-strands, with an N-terminal α-helix. The pore dimensions are roughly 3–4 nm diameter and 4 nm height. The channels can adopt an open state or a closed one, which controls the permeability of the pores. In vivo VDAC opening/closure is supposed to play an important regulatory role to adjust organelle metabolism. In addition to function in the regulation of metabolite transport, VDACs are involved in programmed cell death [1].

In *Arabidopsis thaliana* (*Arabidopsis*), VDACs are encoded by a small gene family of five members: *VDAC1* (At3g01280), *VDAC2* (At5g67500), *VDAC3* (At5g15090), *VDAC4* (At5g57490) and *VDAC5* (At3g49920). The VDAC1–4 proteins have similar sizes (274–276 aa) and similar putative structures. More than 40% identity is found between these four proteins, and VDAC1 and VDAC3 are the most closely related proteins (68% identity) (Figure S1). Different splice variants are proposed for VDAC5, and all resulting protein models are truncated compared to VDAC1–4 [4,5], (http://plants.ensembl.org/index.html).

The level of each protein has been evaluated in *Arabidopsis* mitochondrion [6]. The most abundant VDAC is VDAC1, with an estimated 44,400 copies per mitochondrion [6], followed by VDAC3 (23,200 copies per mitochondrion), VDAC2 (11,400 copies), VDAC4 (1600 copies) and VDAC5 (30 copies). Fuchs et al. have estimated that, altogether, VDACs represent 34% of MOM surface. In comparison, the translocase of the outer membrane (TOM) complex, involved in protein import, represents 12% of MOM area [6]. In addition to MOM localization, several studies have also found non-mitochondrial localizations of VDAC proteins, in particular, in plastids with some contradictory results [1] and in plasma membrane [5].

T-DNA insertion mutant lines in *VDAC1*–*4* have been characterized. The phenotype of knockout (KO) mutant plants is highly variable. *vdac3* plants look like wild-type plants. By contrast, *vdac1*, *vdac2* and *vdac4* mutants present an altered phenotype (smaller rosettes, delayed development and poor seed production), with increasing defects from *vdac1* to *vdac2*, then *vdac4* [4,5]. The *vdac4* phenotype is particularly severe, with stunted plants and dramatically reduced seed production. Moreover, mitochondria appear enlarged in *vdac1* and *vdac4*: nearly 90% of Col0 mitochondria have a surface area less than 0.8 mm^2^, but this size is found in only 30% of *vdac1* and 25% of *vdac4* mitochondria [5]. These observations suggest that the different *VDAC* genes are not strictly redundant. However, the specific functions of each *VDAC* are still unclear.

Here, we show that VDAC4 strongly interacts with tRNAs, so it has probably acquired a specialized function in tRNA import into mitochondria. VDAC1 interacts with tRNA with a lower efficiency, but it does not seem to participate in tRNA import in vivo. *VDCA3* is characterized by an early induction in stress conditions, and both *vdac1* and *vdac3* KO lines are weakly affected in oxidative phosphorylation (OXPHOS), but they cannot rescue each other for this defect. Taken together, this work elucidates distinct and overlapping functions of VDACs in the regulation of mitochondrial metabolism.

## 2. Materials and Methods

### 2.1. Plant Material and Growth Conditions

The insertion mutants SALK_034653 for *vdac1* and SALK_127899.41.10.x for *vdac3* [5] were from the Columbia ecotype. Both lines were kindly provided by Dr. Filleur (I2BC, Gif/Yvette, France). *vdac1*_OEV3 corresponds to a *vdac1* mutant transformed with the VDAC3 genomic sequence (from its promoter to the end of its short 3′ UTR, and with a HA tag at the beginning of the coding sequence (CDS) [7]. *Arabidopsis* cell cultures were from the Landsberg ecotype [8].

Plants were grown in long-day conditions (16 h day at 21 °C/8 h night at 18 °C cycles, LED tubes Philips 1500 mm SO 20W 840 T8, photon flux density of 120 mmol/s/m^2^ at the plant level). Seedling cultures and cell cultures were grown at 23 °C with constant light. Seedlings were grown in Murashige and Skoog MS231 medium (Duchefa Biochemie, Haarlem, The Netherlands) (8 days in hydroponic cultures or 10 days on plates for O_2_ consumption experiments). Cell cultures were grown in Murashige and Skoog MS256 medium.

Stress conditions were performed on 8-day-old cell cultures. Cold and heat shocks were for 3 h at 4 °C or 37 °C, respectively. NaCl (150 mM) and H_2_O_2_ (20 mM) stresses and phosphate starvation were for 24 h at 23 °C. For phosphate starvation studies, media used were MSP01 (with phosphate) and MSP11 (without phosphate) (Caisson Labs, Smithfield, UT, USA).

### 2.2. Cloning, Overexpression and Purification of VDAC Proteins

*Arabidopsis* VDAC1–4 CDSs were amplified by RT-PCR and cloned into pDON207 entry vector and then into p0GWA destination vector [9] using the Gateway recombination cloning technology. Constructs corresponding to potato VDAC34 and VDAC36 have been previously obtained [10]. All CDSs were in fusion with a His tag at the C-terminal end of the protein.

Primers used for PCR (*attB* sequences in italics):
VDAC1(direct)gggg*acaagtttgtacaaaaaagcaggct*tggtgaaaggtcccggtctcVDAC1(reverse)gggg*accactttgtacaagaaagctgggt*aaggcttgagtgcgagagccVDAC2(direct)gggg*acaagtttgtacaaaaaagcaggct*tgagcaaaggtccaggactcttVDAC2(reverse)gggg*accactttgtacaagaaagctgggt*aaggtttgagagcaagagagVDAC3(direct)gggg*acaagtttgtacaaaaaagcaggct*tggttaaaggtccaggactctaVDAC3(reverse)gggg*accactttgtacaagaaagctgggt*agggcttgagagcgagagcVDAC4(direct)gggg*acaagtttgtacaaaaaagcaggct*tgggaagcagtccagctccgVDAC4(reverse)gggg*accactttgtacaagaaagctgggt*atggtttgagggcgagggc

VDAC proteins were overexpressed in *Escherichia coli* and purified with His60 Ni Superflow Resin (Takara #635660, Takara Bio Europe, Saint-Germain en Laye, France) in 1 mL columns under denaturing conditions (8 M urea) according to manufacturer’s recommendations.

### 2.3. Northwestern Experiments

The construct containing *Arabidopsis* cytosolic tRNA^Ala^ gene sequence has been previously obtained [11]. The tRNA gene sequence was bordered with a T7 RNA polymerase promoter in 5′ part and a BstNI restriction site in 3′ part. The construct was used as a substrate for T7 polymerase to synthesize radiolabeled transcripts in vitro.

Northwestern blots were performed as described in [10]. Overexpressed VDAC proteins were electrophoresed on SDS-PAGE and transferred onto Immobilon-P membranes. For renaturation, membranes were incubated in 0.1 M Tris-HCl (pH 7.5) and 0.1% NP40 overnight at 4 °C, then washed 4 times in the same buffer for 15 min at 4 °C. After washing, membranes were incubated in binding buffer (10 mM Tris-HCl (pH 7.5), 5 mM magnesium acetate, 2 mM dithiothreitol (DTT, 0.01% (*v/v*) Triton X-100) with 5% (*w/v*) bovine serum albumin (BSA) for 5 min at 4 °C, then overnight at 4 °C in binding buffer with the in vitro synthetized radiolabeled tRNA^Ala^ transcripts. Washing was performed 4 times for 5 min at 4 °C in binding buffer. For each experiment, signals were detected with an Amersham Typhoon imager and quantified with the ImageJ software (https://imagej.nih.gov/ij/download.html).

### 2.4. Mitochondria Preparation

Mitochondria were prepared from 8-day-old water-cultured seedlings [12]. Seedlings were filtered and ground in a mortar in grinding buffer (0.3 M sucrose, 25 mM tetrasodium pyrophosphate, 2 mM EDTA, 10 mM KH_2_PO_4_, 1% (*w/v*) PVP-40 (polyvinylpyrrolidone), 1% (*w/v*) BSA, 20 mM sodium ascorbate, 20 mM L-cysteine, pH 7.5). Homogenate was filtered in 50 μm nylon meshes and one layer of Miracloth (Merck Millipore, Molsheim, France). Filtered homogenate was centrifuged successively for 5 min at 1600*g* and 2000*g* to eliminate cellular debris. The supernatant was then centrifuged at 17,200*g* for 20 min. The mitochondria pellet was resuspended in 1 mL of washing buffer (0.3 M sucrose, 10 mM MOPS (3-(*N*-morpholino)propanesulfonic acid), pH 7.2) with 1% (*w/v*) BSA, and purified on a continuous PVP-40 0–4.4% (*w/v*) gradient by centrifugation at 40,000*g* for 40 min. The continuous gradient was prepared from 15 mL of heavy gradient solution (4.4% (*w/v*) PVP-40, 32% (*v/v*) Percoll in washing buffer with 1% (*w/v*) BSA) and 15 mL of light gradient solution (32% (*v/v*) Percoll in washing buffer with 1% (*w/v*) BSA). Mitochondria in the bottom of the gradient were washed once in washing buffer with 1% (*w/v*) of BSA and twice in washing buffer without BSA.

To eliminate RNA contaminants at the surface of mitochondria (for northern blots), the purified mitochondria were incubated with RNases (1 μL of Thermo Fisher RNases A/T1 mix per mg of mitochondrial proteins) for 10 min on ice, centrifuged and then total RNA was extracted with Tri-reagent^®^ (Molecular Research Center, Cincinnatti, OH, USA) according to manufacturer’s recommendations.

### 2.5. Northern Blots

RNA was separated on 15% polyacrylamide gel in denaturing conditions and electro-transferred onto a nylon membrane. Hybridizations were performed overnight at 45 °C in 6X SSC and 0.5% (*w/v*) SDS. Washes were for 30 min in 2X SSC and 0.1% SDS [13]. The oligonucleotide probes were labeled with ^32^P using T4 polynucleotide kinase:

- Cytosolic tRNA^Lys^: 5′ CGCCCACCGTGGGGCTCGAACCC 3′
- Imported tRNA^Ala^: 5′ ACCATCTGAGCTACATCCCC 3′
- Imported tRNA^Gly^_CCC_: 5′ TGCGCATCCAGGGAATCGAAC 3′
- Native tRNA^Gly^_GCC_: 5′ AGCGGAAGGAGGGACTTGAACCCTCA 3′

### 2.6. RNA Extraction, Reverse Transcription and RT-qPCR

RNA was extracted from mitochondria and whole cells using TRI Reagent^®^ (Molecular Research Center, Cincinnatti, USA) according to the manufacturer’s instructions, then treated with RNase-free Dnase RQ1 (Promega, Fitchburg, WI, USA) and quantified with NanoDrop. RNA quality was checked by electrophoresis in MOPS buffer/formaldehyde/agarose gel. Reverse transcription was performed with Reverse Transcription SuperScript™ III (Invitrogen, ThermoFisher Scientific, Waltham, MA, USA in the presence of hexamers and oligo-dT primers [14]. For qPCR, the RT was used directly and diluted 10 times, and 2–3 technical replicates were performed for each dilution. Primers are listed in Table 1. At least three biological replicates, corresponding to mitochondrial and total RNA extracted from the same material, were prepared. The qPCR efficiency for each primer couple was determined by serial dilutions of cDNA. The qPCR results were normalized with cytosolic ribosomal protein L12 (RPL12) or glyceraldehyde-3-phosphate dehydrogenase (GAPDH) mRNAs.

### 2.7. O_2_ Consumption

Oxygen consumption of seedlings was measured in 2 mL of deionized water with a liquid-phase Oxytherm oxygen electrode system (Hansatech Instruments, Pentney, UK). Ten-day-old seedlings grown on plates (30–40 mg) were directly imbibed in the electrode chamber and rates of oxygen consumption were measured. After 6 min, KCN was added (final concentration: 2.5 mM) and oxygen consumption was re-measured. Differences between these two rates corresponded to cyanide-sensitive oxygen uptake (OXPHOS O_2_ uptake) (*N* = 3–5).

### 2.8. Proteomics

Two independent mitochondrial preparations (see above) were obtained from each plant line. Each preparation was then split into two or three parts. Proteins (1 mg) were extracted from each part, digested with trypsin and then analyzed by nanoLCMS/MS. Data were searched against the TAIR *A. thaliana* database. All steps were performed at the “Plateforme Protéomique Strasbourg-Esplanade” (http://www-ibmc.u-strasbg.fr/proteo/Web/accueil.htm).

To identify significantly affected proteins, a statistical analysis based on spectral counts was performed using a homemade R package (https://www.r-project.org/) as described in [15], except that the size factors used to scale samples were calculated according to the DESeq2 normalization method (i.e., median of ratios method, [16]). The R package performs a negative binomial test using an edgeR-based GLM (Generalized Linear Models) regression [17], and calculates the fold change and an adjusted *p*-value corrected by Benjamini–Hochberg for each identified protein.

Mitochondrial localization was determined according to SUBA4 (http://suba.live/). For functional annotations (see Table S2), proteins were assigned as tricarboxylic acid cycle (TCA) enzymes according to [18] (class no. 1), OXPHOS according to [19] (class no. 2), or RNA process and translation according to [19,20,21] (class no. 3).

### 2.9. Western Blot and Antibodies

A western blot analysis was conducted according to the standard protocol. Antibodies against the ribosomal rPPR1 protein (At1g64870) and the alternative oxidase (AOX) were from P. Giegé (IBMP, Strasbourg, France) [22] and T. Elthon (GT monoclonal antibodies, University of Nebraska, Lincoln, NE, USA) [23], respectively.

## 3. Results

### 3.1. VDAC4 Strongly Interacts with tRNAs

Plant mitochondria have the particularity to import numerous tRNAs (one third to one half of tRNA species) [24]. The mechanism of import has been elucidated at the level of MOM, involving both TOM and VDAC. TOM appears to be implicated in the binding of tRNAs at the mitochondrial surface, and VDAC in the translocation step through MOM [3]. *Solanum tuberosum* (potato) mitochondria contain two major VDAC proteins, VDAC34 and VDAC36. The two proteins were shown to differentially interact with tRNAs in vitro, with VDAC34 being the more efficient [10]. By comparing the binding efficiency of wild-type and mutated VDACs, a few positions were shown to be mandatory for the interaction, even if a clear binding site could not be identified.

**Figure 1:**
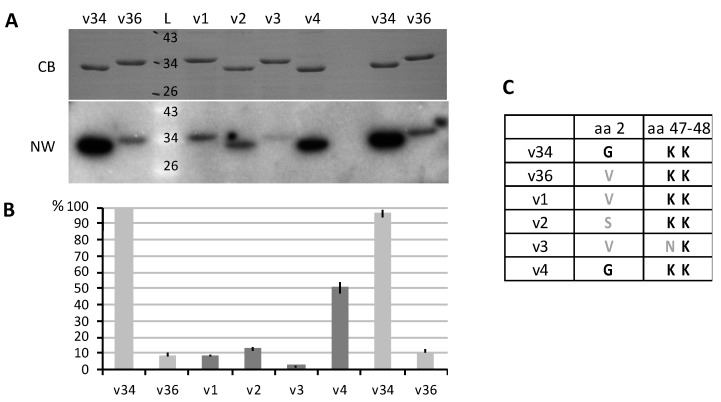
Voltage-dependent anion channel (VDAC) proteins differentially interact with tRNAs in vitro. The six VDAC proteins, potato VDAC34 and VDAC36 (v34, v36) and *Arabidopsis* VDAC1–4 (v1, v2, v3 and v4) were overexpressed with a His tag in *Escherichia coli* and purified. The purified proteins were separated on SDS/PAGE and transferred onto membranes. (**A**) Northwestern (NW) blots with radioactive tRNA^Ala^ transcripts. CB, Coomassie blue staining of the membrane; L, ladder (in kDa). (**B**) Quantification. Three similar blots were hybridized with tRNA transcripts. The signals were quantified and normalized on the first v34 loading, and means were calculated. (**C**) Amino acids in positions 2, 47 and 48. Gly_2_ and K_47_K_48_ have been shown to be involved in tRNA interaction [10]. Error bars, standard error of the mean (SEM).

In order to evaluate VDAC–tRNA interactions, *Arabidopsis* VDAC1–4 and potato VDAC34 and VDAC36 proteins were overexpressed in *Escherichia coli*. Northwestern experiments were performed to detect the direct binding of tRNA^Ala^ transcripts to purified VDACs immobilized on a membrane (Figure 1). As already shown, the signal obtained with VDAC34 was far higher than that obtained with VDAC36 [10]. Concerning *Arabidopsis* VDACs, a strong signal was observed with VDAC4, moderate ones with VDAC1 and VDAC2, and a very weak one with VDAC3. Compared to VDAC4, signals with VDAC1 or VDAC2 were about 5 times lower, and that with VDAC3 were about 20 times lower. Thus, *Arabidopsis* VDACs appeared to differentially interact with the tRNA transcripts, suggesting a functional specialization of these proteins.

The binding of tRNAs to VDAC4 appeared to be half of that observed for VDAC34. In VDAC34, three amino acids were shown to be essential for the interaction, a glycine residue in position 2 and two lysine residues in positions 47 and 48. The mutation of Gly_2_ in VDAC34 reduced the interaction to half, and the mutation of the two lysine residues induced a loss of 2/3 of the interaction [10]. These three positions are conserved in VDAC4, so the difference in binding should be linked to other sequence differences. The two lysine residues are also found in VDAC1, VDAC2 and VDAC36, but not Gly_2_. One lysine residue and Gly_2_ are not present in VDAC3 (Figure 1C and Figure S1). These differences could explain the moderate binding of VDAC1, VDAC2 and VDAC36, and the weak binding of VDAC3.

VDAC4 appeared to strongly interact with tRNA in vitro. The *vdac4* KO plant phenotype is very strong (Figure S2). Hence, it was not possible to prepare highly purified mitochondria from this material. As VDAC1 moderately interacts with tRNA, but is also the major VDAC in MOM [6], its role in tRNA import into mitochondria was evaluated. For that, three *Arabidopsis* lines were used, the wild-type Col0, a *vdac1* KO mutant that presents altered growth, and the *vdac1* mutant overexpressing VDAC3 (*vdac1*_OEV3). This *vdac1*_OEV3 line corresponds to a *vdac1* mutant transformed with the VDAC3 genomic sequence, from its promoter to the end of its short 3′ UTR [7] (see below). The phenotype of *vdac1*_OEV3 plants is similar to that of wild-type plants (Figure S2), as is the size of their mitochondria [7]. A proteomic analysis of mitochondria (see Section 3.3.) showed that the steady state levels of VDAC2 and VDAC4 were similar in the three lines, and that VDAC3 level was similar in wild-type (WT) and *vdac1,* and roughly twice higher in *vdac1*_OEV3 (Figure S2).

**Figure 2:**
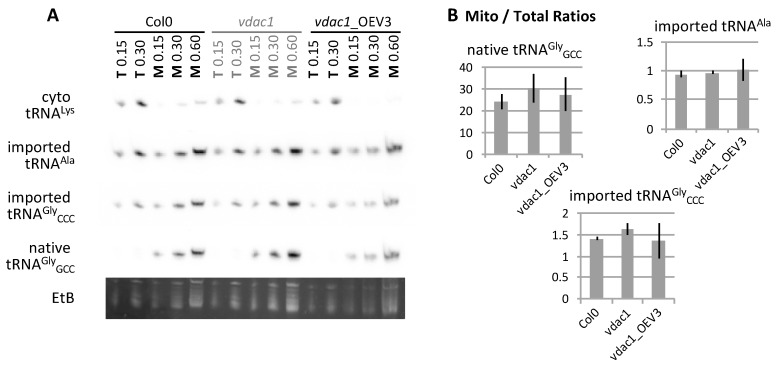
In vivo import of tRNAs into mitochondria. Mitochondria were extracted from Col0, *vdac1* and *vdac1*_OEV3 seedlings. Total (T) and mitochondrial (M) tRNAs were prepared and loaded onto a urea-polyacrylamide gel (0.15–0.60 μg). (**A**) Northern blots. Four tRNA probes were used. The native tRNA^Gly^ corresponds to a mitochondrial-encoded tRNA. The tRNA^Ala^ and tRNA^Gly^_CCC_ are nuclear-encoded and imported into mitochondria. The tRNA^Lys^ is nuclear-encoded, but restricted to the cytosol [24]. This last probe shows that the level of cytosolic contamination is low in mitochondria from every plant line. EtB, ethidium bromide staining. (**B**) Quantification. Two similar blots were performed and the signals were quantified. The M/T ratios were calculated for loadings of 0.15 and 0.3 mg of total and mitochondrial RNAs, respectively. Imported tRNA^Ala^ and native tRNA^Gly^, *N* = 4; imported tRNA^Gly^, *N* = 2. Error bars, SEM.

Mitochondria were prepared from Col0, *vdac1* and *vdac1*_OEV3 lines. After purification on a Percoll gradient, mitochondria were treated with RNAse to eliminate residual RNA at their surface. Total and mitochondrial tRNAs were extracted, and northern blots were performed. The mitochondria/total ratio was determined for a mitochondrial-encoded tRNA (native tRNA^Gly^_GCC_), and two imported tRNAs (tRNA^Ala^ and tRNA^Gly^_CCC_) (Figure 2). No clear difference could be observed between the three plant lines, suggesting that the deletion of VDAC1 did not affect the import of tRNAs into mitochondria.

### 3.2. A VDAC3 mRNA Isoform is Specifically Induced in Stress Conditions

VDACs are at the interface between mitochondria and cytosol, and are supposed to adjust exchanges between these two compartments in response to cell signals and stresses. Moreover, VDAC1 and VDAC3 have been independently found to be induced by stresses [25,26]. According to information found in the Bio-Analytic Resource for Plant Biology (https://bar.utoronto.ca/efp/cgi-bin/efpWeb.cgi), we evaluated the impact of different stresses on VDAC expression in WT cell cultures.

**Figure 3:**
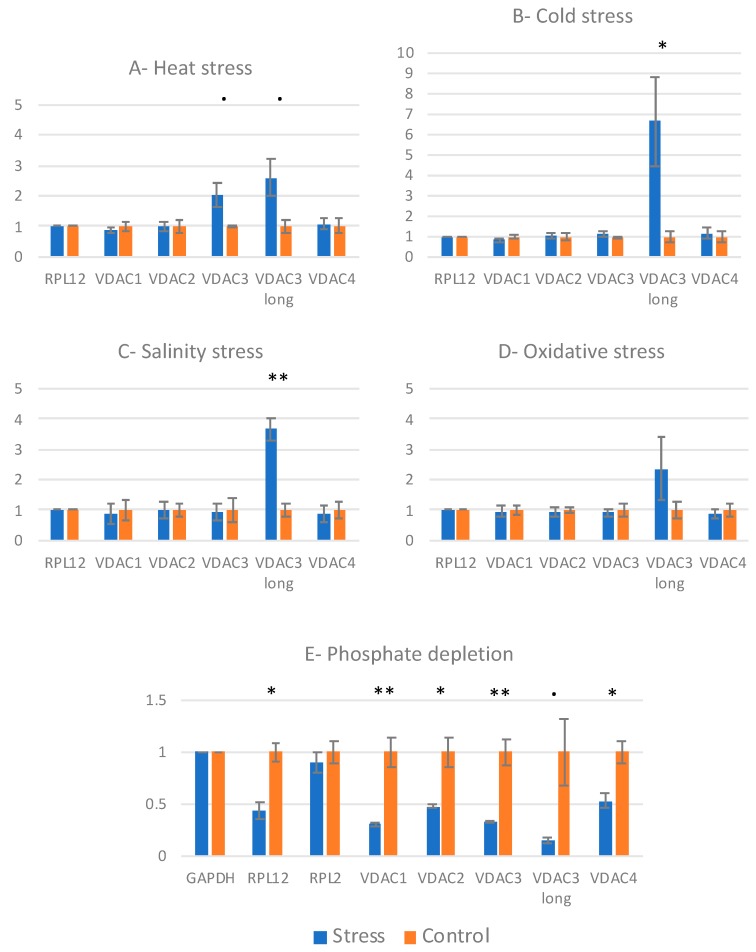
VDAC response to stress. Stress conditions, performed on 8-day-old cell cultures, were 3 h at 37 °C or 4 °C for heat (**A**) and cold stress (**B**), respectively, or 24 h at 23 °C for salinity (150 mM NaCl) (**C**) and oxidative (20 mM H_2_O_2_) (**D**) stresses and for phosphate depletion (**E**). Total RNAs were extracted and used for RT-qPCR. Normalization was done on RPL12 mRNA (**A**–**D**), except for phosphate depletion (**E**). In that case, RPL12 messenger was found downregulated and normalization was done on GAPDH. Error bars, SEM. Student’s test *p*-value: **·** between 5% and 10%, * between 1% and 5%, ** below 1%.

Cold (4 °C) and heat (37 °C) shocks were performed for 3 h, and salt and oxidative stresses for 24 h. Total RNA was then extracted and VDAC mRNAs were quantified by RT-qPCR (Figure 3). Primers hybridizing with VDAC1, VDAC2 and VDAC4 CDSs were used for qPCR. For VDAC3, identical proteins have been shown to be translated from two mRNA isoforms, which differ by their 3′ UTR and localization in the cell [7]. The major mRNA isoform, which has the shorter 3′ UTR, is cytosolic. The long mRNA, which has a longer 3′ UTR and is about 100 times less abundant than the short one, is associated with the mitochondrial surface [7]. Two couples of primers were used. The first one (VDAC3) recognizes the two isoforms, and the second one (VDAC3 long) only hybridizes with the long isoform (hybridization with the 3′ extension).

None of the stress conditions affected VDAC1, VDAC2 and VDAC4 mRNA levels. VDAC3 total mRNA (the short isoform in majority) was weakly increased in response to heat. More interestingly, VDAC3 long mRNA isoform (with the long 3′ UTR) was found overexpressed in many conditions (2- to 6-fold increase), suggesting that this isoform is specifically involved in stress response (Figure 3A–D).

Phosphate starvation was also performed for 24 h (Figure 3E). The consequence was dramatic for all VDAC mRNAs, with a 50–85% reduction of mRNA levels, but also for other messengers such as RPL12, suggesting a global stress in the cell.

### 3.3. Mitochondrial Proteomes and OXPHOS Respiration

To evaluate the role of the abundant VDAC1, the mitochondrial proteome of the *vdac1* KO line was determined. Mitochondria were thus prepared from *vdac1* and Col0 lines. As the phenotype of *vdac1*-OEV3 plants is normal, mitochondria were also prepared from *vdac1*_OEV3 and *vdac3* lines, to explore which functions could be complemented by VDAC3.

**Figure 4:**
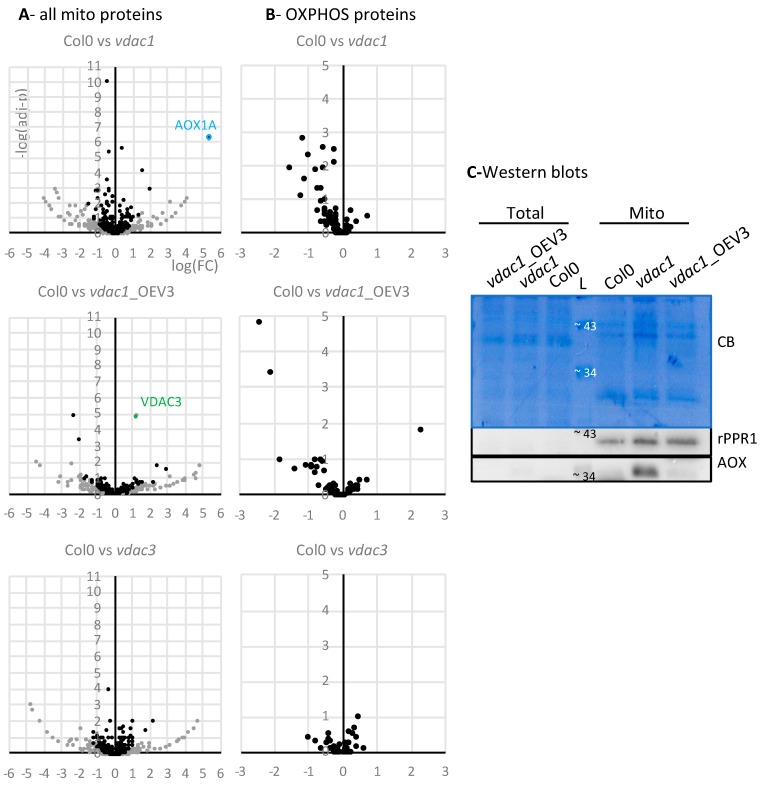
Mitochondrial proteomes. Proteins from purified mitochondria were analyzed by MS/MS, and spectral count label-free quantifications were performed relative to Col0. In volcano plots, the fold change is presented on the *x*-axis (log_2_(FC)) and the adjusted *p*-value on the *y*-axis (–log_10_(adj-*p*)). (**A**) Mitochondrial proteins. Due to their high *x* and *y* values, VDAC1 is not shown on graphs corresponding to *vdac1* and *vdac1*_OEV3, and VDAC3 on graph corresponding to *vdac3* (Figure S3). Plot scales were adjusted according to the other proteins. Proteins with a mean of less than three spectra in the most expressed condition are in grey. They will not be considered in (**B**) and Figure S3. (B) OXPHOS proteins, according to [19]. (**C**) Western blots with antibodies against the ribosomal protein rPPR1 and AOX. Proteins at 10 μg were loaded in each lane. CB, Coomassie blue staining; L, ladder.

Mass spectrometry analyses identified about 700 mitochondrial proteins (according to SUBA4 annotation) in each mitochondrial extract. The total number of MS/MS fragmentation spectra was then used for label-free quantification, comparing *vdac1* to Col0. For each protein, a protein fold-change (FC) and an adjusted *p*-value (adj-*p*) were calculated. Similar quantifications were also performed with *vdac1*_OEV3 and *vdac3* mitochondria, compared to Col0 (Figure 4 and Figure S3).

In all three comparisons, only a few proteins were identified as significantly enriched or depleted (i.e., with adjusted *p*-value < 0.1) in each *vdac* line compared with Col0 (Table S2, Sheet 6). VDAC1 and VDAC3 were of course among them (VDAC1 underexpression in *vdac*1 and *vdac1*_OEV3, and VDAC3 underexpression in *vdac*3 and overexpression in *vdac1*_OEV3). It should be noted than FC and adj-*p* value should be considered with caution for proteins with a low spectral count. To avoid too much bias, only proteins with at least three spectra in the most expressed condition were further considered. With these criteria (adjusted *p*-value < 0.1; spectral count > 3), 30 and 19 proteins were found to be underexpressed and overexpressed in *vdac1* mitochondrial proteome, respectively. In *vdac1*_OEV3 and *vdac3*, 4 and 10 proteins were underexpressed, and 3 and 14 proteins were overexpressed, respectively.

Functional annotation clustering (DAVID Bioinformatics Resources 6.8; https://david.ncifcrf.gov/) of the *vdac1* underexpressed proteins pointed out functional groups such as “oxidative phosphorylation”, “complex I” and “mitochondrion membrane”, and no convincing enrichment scores were obtained in other lines or conditions (under/over). This clustering suggested that some proteins involved in oxidative phosphorylation (OXPHOS) could be affected in *vdac1*. Hence, OXPHOS proteins were analyzed more deeply. In *vdac1*, compared to Col0, 53 out of the 73 OXPHOS proteins had a negative logFC, with an adj-*p* value below 0.1 for 11 of the proteins (Figure 4B, –log(adj-*p*) > 1) (Table S1, Sheet 3). This bias was not observed in *vdac1*_OEV3 or *vdac3*, and in other pathways such as the TCA cycle, RNA processing and translation (Figure S3). Among the 11 underexpressed OXPHOS proteins in *vdac1*, 6 proteins were from Complex I, 4 proteins were from Complex V and 1 protein was from Complex II. These results suggested that many OXPHOS proteins were weakly affected in *vdac1* line, and restored in *vdac1*_OEV3.

In addition, four proteins involved in ATP transport through the inner membrane and identified in the same complex [19] were also found underexpressed in *vdac1*, but not in other lines (Table S2, Sheet 6). The alternative oxidase AOX1a was found as the most overexpressed protein in *vdac1*, and its overexpression was confirmed with a western blot (Figure 4C). AOX1a is usually considered as an indicator of stress and mitochondria dysfunctions [5,27,28], which is in accordance with the altered phenotype of *vdac1* mitochondria and plants.

**Figure 5:**
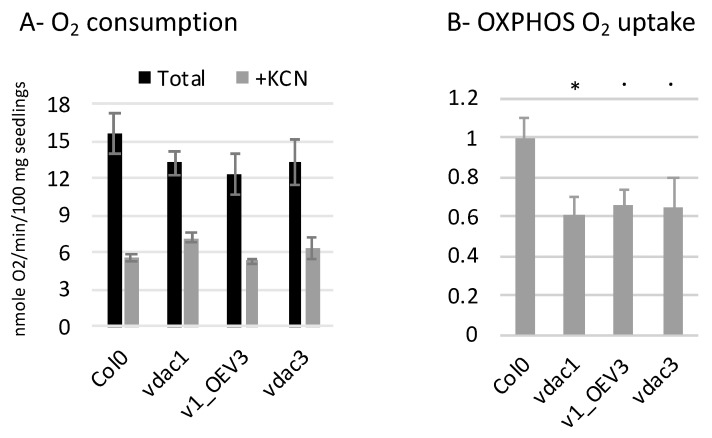
O_2_ uptake in seedlings. The oxygen consumption was measured on seedlings (30–40 mg) with an oxygen electrode system. After a first recording of 6 min, KCN, which blocks OXPHOS activity, was added. (**A**) Total oxygen consumption, and after KCN addition (nmol O_2_/min/100 mg seedlings) (mean values of 3–5 assays). (**B**) OXPHOS O_2_ uptake was determined by subtracting residual KCN consumption to total, and expressed relatively to Col0. Student’s test *p*-value: · between 5% and 10%, * between 1% and 5%. Error bars, SEM.

To evaluate OXPHOS function, oxygen uptake was then measured in seedlings from Col0, *vdac1*, *vdac1*_OEV3 and *vdac3* lines. The total O_2_ consumption was not strongly affected in the mutant lines compared to Col0. However, O_2_ uptake by the OXPHOS pathway was clearly reduced in *vdac1*, and also in *vdac1*_OEV3 and *vdac3* (Figure 5A,B).

## 4. Discussion

Four VDACs are found in *Arabidopsis* MOM and all of them are expected to exchange metabolites between cytosol and mitochondria. However, the *vdac* KO phenotypes are clearly distinct, and do not correlate with VDAC protein abundance observed in WT cells [5,6]. This suggests that VDAC proteins have specific functions.

In order to identify the functions of the different VDACs, we first explored their capacity to interact with tRNAs. The in vitro test showed that the four VDAC proteins had different behaviors. VDAC4 was found to be the strongest tRNA interactant, which suggested that VDAC4 could be important in tRNA import into mitochondria.

VDAC1 moderately interacts with tRNAs and is highly abundant in MOM, but it has not been found essential for in vivo import of tRNAs into mitochondria. VDAC2 interaction with tRNA is similar to that of VDAC1, but VDAC2 is less abundant than VDAC1 in MOM [6], so it is unlikely that it plays an important role in tRNA import into mitochondria. VDAC3 very weakly binds to tRNAs. Two others mitochondrial proteins, Tric1 and Tric2, (tRNA import components) have been found to interact with tRNAs but, as for *vdac1*, the steady state level of imported tRNAs does not seem affected in mitochondria of *tric1 tric2* double KO line [29]. Thus, VDAC4 remains the best candidate for a tRNA channel in MOM. If tRNA import is strongly impaired, the mitochondrial translation would also be affected and in consequence, the whole plant metabolism. This is in accordance with the very strong phenotype of vdac4 plants.

Second, VDAC response to stress was investigated. Phosphate (Pi) starvation induced a repression of all VDAC mRNAs, but also of other mRNAs. Pi-starved plants are known to extensively remodel their transcriptome and proteome to coordinate metabolic and morphological adaptations. Misson et al. have shown the induction and suppression of 612 and 254 Pi-responsive genes, respectively, in *Arabidopsis* [30]. These genes are involved in transcriptional regulation, ion transport and various metabolic pathways. Indeed, alternative pathways for cytosolic glycolysis and mitochondrial electron transport promote the survival of Pi-deprived plants [31]. The effect of Pi starvation on VDACs is thus not surprising.

Other tested stresses were found to specifically induce the VDAC3 long mRNA isoform. Abiotic and biotic stresses [25,26] have already been shown to induce VDAC proteins, in particular, VDAC1 or VDAC3, but the results have been sometimes contradictory [32]. One explanation could be that the growth and stress conditions (in particular, time) were not identical, as well as the plant material (seedlings, mature plants, etc.). In our experiments, we used cell cultures and observed that the VDAC3 long mRNA isoform responded to many stresses. This long mRNA isoform has been shown to be targeted to the mitochondrial surface, thanks to its 3′ extension [7]. Targeting of cytosolic mRNAs to the mitochondrial surface has been demonstrated in various organisms, and a co-translational pathway for protein import is now proposed in parallel to the conventional post-translational model [33]. VDAC3 mRNA targeting does not change the efficiency of VDAC3 protein import [7]. A hypothesis for VDAC3 mRNA localization at MOM is that it could favor a specific localization of the protein into particular MOM microdomains or specific interactions with other proteins, similar to that proposed for prolamin and glutelin mRNAs at the endoplasmic reticulum [34]. VDAC3, more than any other VDAC, has been shown to interact with different proteins such as glycolytic enzymes [35,36], proteins involved in ROS response [26,37] or proteins associated with cytoskeleton [38]. Interactions with glycolytic enzymes would favor the channeling of metabolites to mitochondria, but these interactions appear to be redox-dependant. Wojtera-Kuritz et al. and Schneider et al. have proposed that these interactions would be involved in adaptation to oxidative stress [36,39]. It is thus tempting to propose that stress, by inducing VDAC3 long mRNA, would induce a quick translation of VDAC3 at MOM, thus favoring its interaction with glycolytic enzymes or other proteins, and finally a quick response to stress.

Last, O_2_ uptake by the OXPHOS pathway was reduced in *vdac1*, and also probably in *vdac3*. Reduced ATP synthesis and reduced membrane potential have already been observed in *vdac1* mutants [4,40], and reduced membrane potential has been found in *vdac3*, but also in *vdac2* and *vdac4* KO lines [4]. VDAC3 overexpression did not seem able to restore OXPHOS respiration in *vdac1*_OEV3, suggesting that VDAC1 and VDAC3 have some specialized functions that do not overlap. On the other hand, the overexpression of VDAC3 in *vdac1* background restored OXPHOS protein level, mitochondria size and plant phenotype (this work, [7]), suggesting that VDAC1 and VDAC3 also have some overlapping functions.

Similarly, plant phenotype of *vdac1* can be restored by the overproduction of VDAC4 [5]. Robert et al. [5] have also crossed *vdac1* and *vdac4* KO mutants. They have obtained double heterozygous F1 plants (i.e., with one WT and one mutant allele at each locus), but no double homozygous F2 plants from self-fertilization of this F1. This suggests that VDAC1 and VDAC4 share functions that are essential for plant viability.

In conclusion, VDACs have partially redundant functions in mitochondria, but they are not fully interchangeable pores at MOM, and each VDAC seems to also have its own specificity. This work brings new knowledge in these particular functions, even if further investigations are clearly needed to decipher all these functions.

## Figures and Tables

**Table 1:** qPCR primers.

Gene	Efficiency	Direct/Reverse	Sequence (5′ to 3′)
*RPL2*	1.945	Direct	CCGAAGACGGATCAAGGTAA
*(mito)*		Reverse	CGCAATTCATCACCATTTTG
*GAPDH*	1.967	Direct	AGGCTGCTGCTCACTTGAA
*(At1g13440)*		Reverse	AACATGGGCGCATCTTTG
*RPL12*	1.887	Direct	GACGTGTACGTCCGAGTAACC
		Reverse	GACCGATTTTGGGAGCTAGA
*VDAC3 long*	2.008	Direct	TCCATATCTTTTACTTGGTTCTCTCTT
		Reverse	GGGAACTCCAAATGGAACAA
*VDAC3*	1.974	Direct	CACTGAAATCGGCAAAAAGG
		Reverse	TGTTCCCGTTGTAGTGATCG
*VDAC1*	1.984	Direct	GCTCTTGTTCTTTCGATTCTCAG
		Reverse	TGGTCACTGTTGTGGTCTTTG
*VDAC2*	1.974	Direct	CCGATCTCTCTCAATCTCCG
		Reverse	AGTCTCTCGTCAACAGATCTTTGG
*VDAC4*	1.914	Direct	CGTCGATCTTCCATTTTCG
		Reverse	AATCCTTGTTTAGGAGATCTTTGG

## Supplementary Materials

The following are available online at https://www.mdpi.com/2073-4409/9/4/1023/s1, Figure S1: *Arabidopsis* VDAC sequence similarities and identities, Figure S2: Col0 and *vdac* KO mutants. (**A**) Phenotypes of plants that were 7–8 weeks old. As already shown [4,5,7], *vdac4* phenotype is dramatic. *vdac3* line looks like wild-type. *vdac1* plants are smaller with a delayed development, but *vdac1*_OEV3 plants are normal. (**B**) VDAC protein levels in the mitochondria of Col0, *vdac1* and *vdac1*_OEV3. The spectral counts obtained from proteomic analyses were normalized according to Col0, Figure S3: Mitochondrial proteomes. Proteins from purified mitochondria were analyzed by MS/MS, and spectral count label-free quantifications were performed relative to Col0. Fold change (FC) and adjusted *p*-value (adj-*p*) were calculated. Due to their high FC and adj-*p* values (**A**), VDAC1 and VDAC3 are not shown on graphs, and scales are adjusted according to the other *x* and *y* values. (**B**) TCA enzymes and (**C**) proteins involved in RNA processing and translation. The adjusted *p*-value is presented on the *y*-axis (log_10_(adj-*p*)) and the fold change on the *x*-axis (log_2_(FC)). Only proteins with a mean of more than three spectra in the most expressed condition were considered, Figure S4: Raw data and quantifications, Table S1: Proteomics: raw data and differential analysis. Sheet 1: raw data, Sheets 2–5: differential analyses of mitochondrial proteins in *vdac* lines compared to Col0, Sheet 6: proteins significantly affected in *vdac* lines (adj-*p* value below 0.1; at least three spectra in the most expressed condition), Sheet 7: number of identified proteins in each pathway.
