# Supplementary material for: Arabidopsis Voltage-Dependent Anion Channels (VDACs): Overlapping and Specific Functions in Mitochondria
Source: Cells. 2020 Apr 21;9(4):1023. doi: 10.3390/cells9041023 (PMC7226135; doi:10.3390/cells9041023)

Figure S1: Sequences of *At*VDAC1-4 (A) and comparison with *St*VDAC34 and -36 (B)

The red stars correspond to positions that are essential for tRNA interaction

A

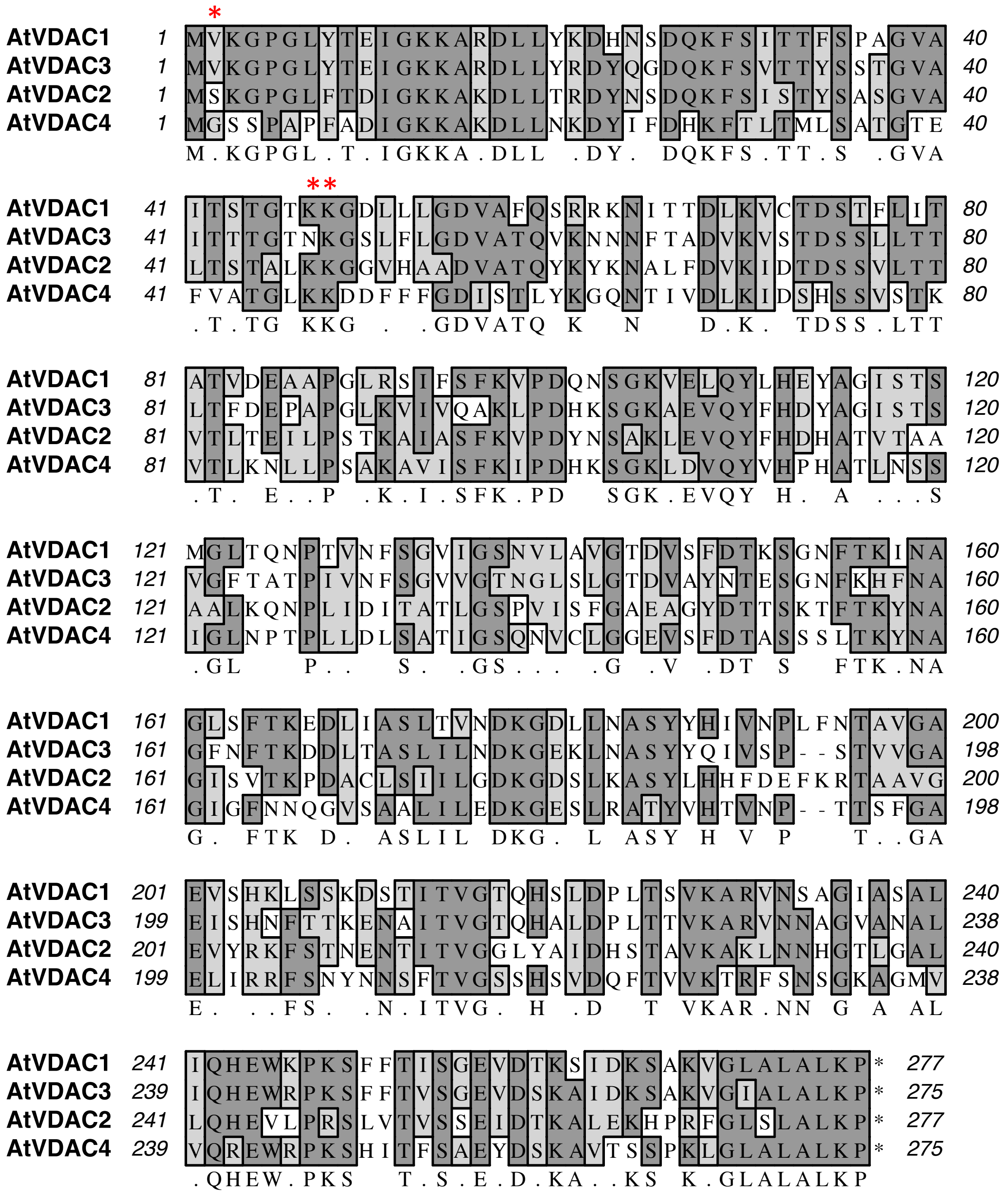

B

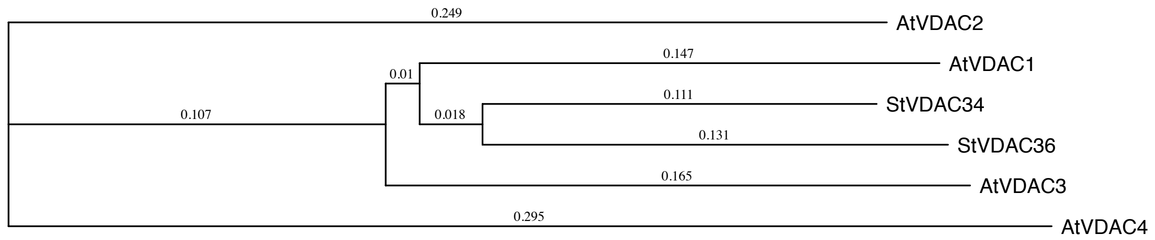

% similarity

%	VDAC34	VDAC36	VDAC1	VDAC3	VDAC2	VDAC4
VDAC34	100	92	86	82	68	65
VDAC36	76	100	86	83	68	68
VDAC1	72	71	100	83	69	63
VDAC3	69	66	68	100	66	62
VDAC2	52	48	50	49	100	65
VDAC4	46	47	42	43	46	100

% identity

Figure S2 : Col0 and *vdac* KO mutants

A- Phenotypes of 7-8 weeks old plants. As already shown[4,5,7], *vdac4* phenotype is dramatic. *Vdac3* line looks like wild type. *Vdac1* plants are smaller with a delayed development, but *vdac1*_OEV3 plants are normal.

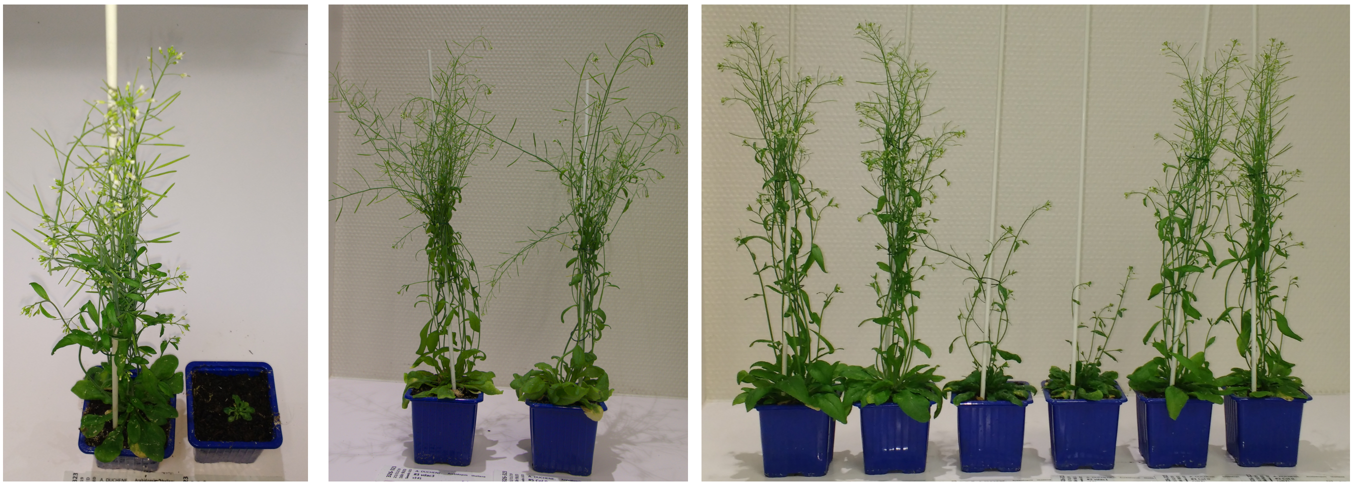

Col0 *vdac4* *vdac3* Col0 *vdac1*_OEV3 *vdac1* Col0

B- VDAC proteins level in mitochondria of Col0, *vdac1* and *vdac1*_OEV3 plant lines.
The mean spectral count for each protein was obtained from proteomic analyses, and was normalized according to Col0. The KO of VDAC1 and VDAC3 did not induce a change in expression of other VDACs.

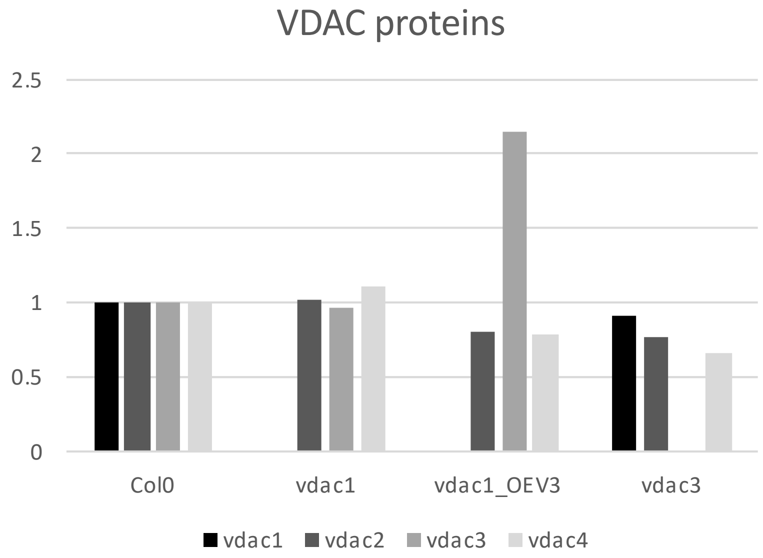

Figure S3: Mitochondrial proteomes

Proteins from purified mitochondria were analyzed by MS/MS, and spectral count label-free quantifications were performed relative to Col0. Fold change (FC) and adjusted p-value (adj-p) were calculated.

(**A**) log_2(FC) and -log_{10}(adj-p) values for VDAC1 and VDAC3 that are not shown on graphs in Fig. 4

(**B**) TCA enzymes and (**C**) proteins involved in RNA processing and translation. The adjusted p-value is presented on y-axes (-log_{10}(adj-p)), and the fold change on x-axes (log_2(FC)). Only proteins with a mean of more than 3 spectra in the most expressed condition are considered.

A-

protein	AGI	plant line	logFC	-log(adjp)
VDAC1	AT3G01280.1	vdac1	-9.9228696	180.881798
VDAC1	AT3G01280.1	vdac1_OEV3	-10.148913	184.646298
VDAC3	AT5G15090.1	vdac3	-9.5751509	136.144034

B- TCA enzymes

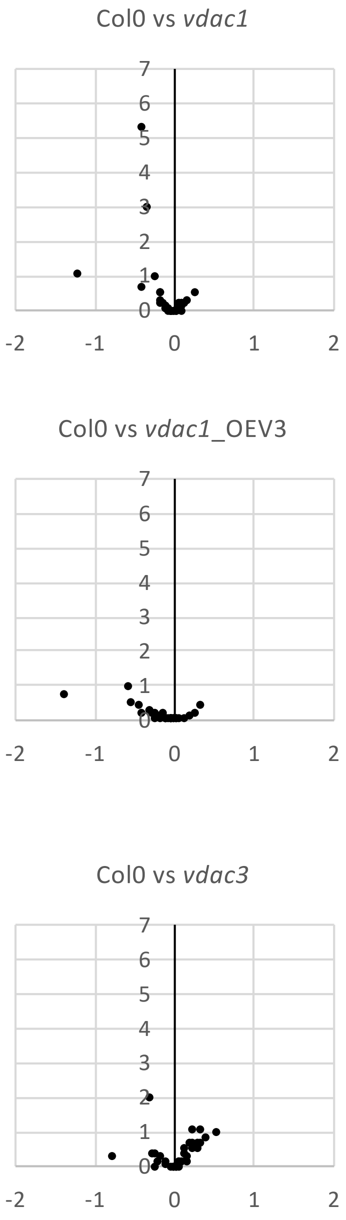

C- Proteins involved in RNA processing and translation

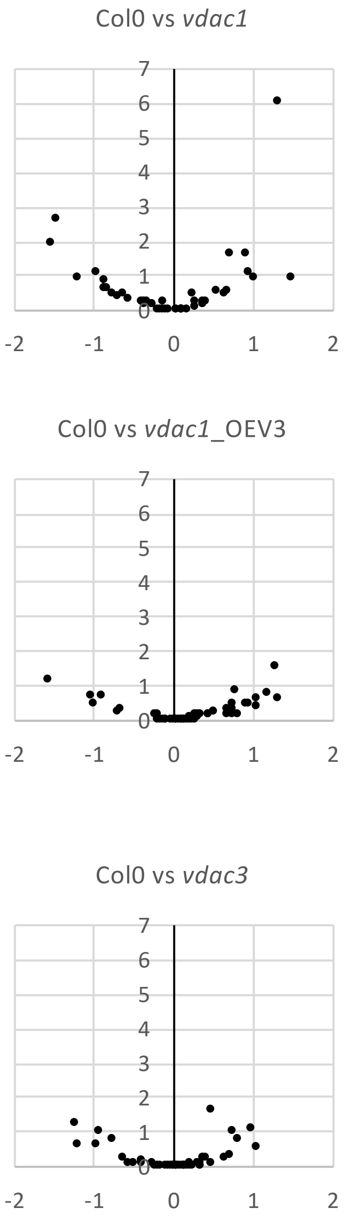

Supplement: Supplementary file 1 [file cells-09-01023-s001.zip › HemonoSupInfo/Hemono_FigS1 to S3.pdf]
