# Supplementary material for: Arabidopsis Voltage-Dependent Anion Channels (VDACs): Overlapping and Specific Functions in Mitochondria
Source: Cells. 2020 Apr 21;9(4):1023. doi: 10.3390/cells9041023 (PMC7226135; doi:10.3390/cells9041023)
Supplement: Supplementary file 1 [file cells-09-01023-s001.zip › HemonoSupInfo/Hemono_FigS4 raw data.pdf]

Raw data for Figure 1: Northwestern blots

A- Coomassie blue staining of the membrane

B- Northwestern blots with radioactive tRNA^{Ala} transcript

Membrane 2

St-VDAC34 St-VDAC36 At-VDAC1 At-VDAC2 At-VDAC3 At-VDAC4 St-VDAC34 St-VDAC36

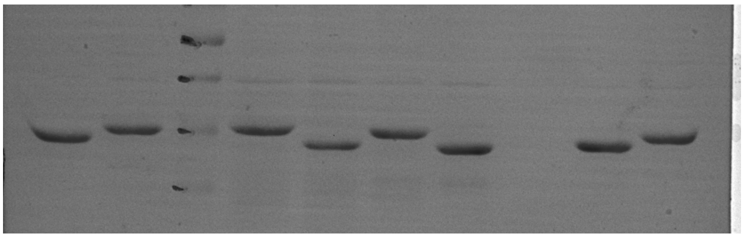

St-VDAC34 St-VDAC36 At-VDAC1 At-VDAC2 At-VDAC3 At-VDAC4 St-VDAC34 St-VDAC36v

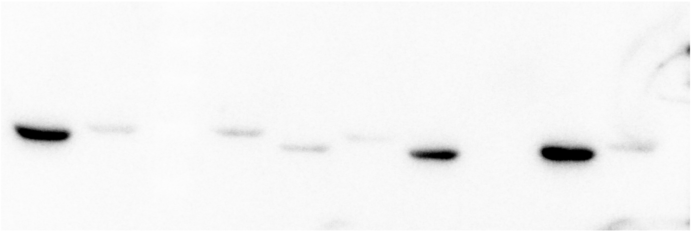

Membrane 3

St-VDAC34 St-VDAC36 At-VDAC1 At-VDAC2 At-VDAC3 At-VDAC4 St-VDAC34 St-VDAC36

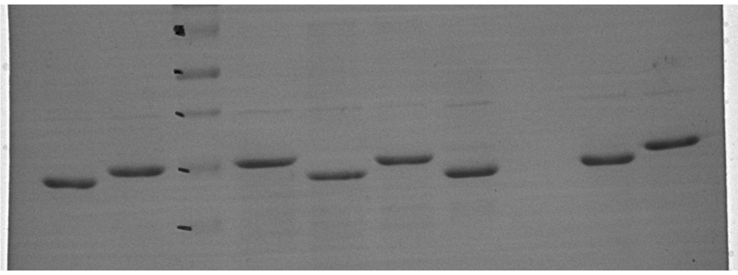

St-VDAC34 St-VDAC36 At-VDAC1 At-VDAC2 At-VDAC3 At-VDAC4 St-VDAC34 St-VDAC36

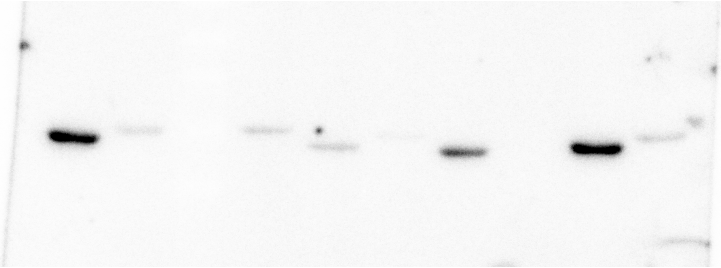

Membrane 4

St-VDAC34 St-VDAC36 At-VDAC1 At-VDAC2 At-VDAC3 At-VDAC4 St-VDAC34 St-VDAC36

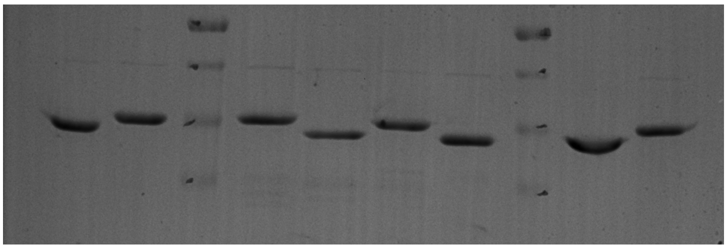

St-VDAC34 St-VDAC36 At-VDAC1 At-VDAC2 At-VDAC3 At-VDAC4 St-VDAC34 St-VDAC36

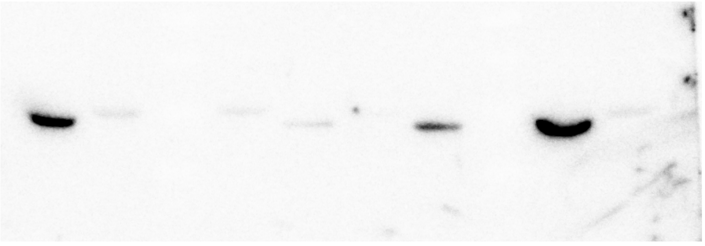

C- quantification

membrane	sample	lane	Coomassie staining of membrane: quantification (arbitrary units)	tRNA-Ala radioactive signal: quantification (arbitrary units)
2	v34	1	2704.9	32671.5
2	v36	2	2236.0	1967.2
2	v1	3	2495.9	2947.9
2	v2	4	2055.4	2474.4
2	v3	5	2363.9	1077.7
2	v4	6	2923.1	20955.6
2	v34	7	3009.9	38094.5
2	v36	8	2595.6	2915.7
3	v34	1	2476.4	30075.5
3	v36	2	2706.0	2033.4
3	v1	3	2420.2	2394.1
3	v2	4	2005.4	3242.5
3	v3	5	2392.1	566.3
3	v4	6	2846.2	13672.6
3	v34	7	2787.8	31684.4
3	v36	8	2722.3	2941.0
4	v34	1	2452.3	31305.2
4	v36	2	2231.5	3817.4
4	v1	3	2362.2	2459.3
4	v2	4	1692.4	3271.0
4	v3	5	2547.0	448.9
4	v4	6	2730.1	18311.6
4	v34	7	3709.6	42541.9
4	v36	8	2364.5	4524.7

Raw data for Figure 2: Northern blots

Membrane NB02:

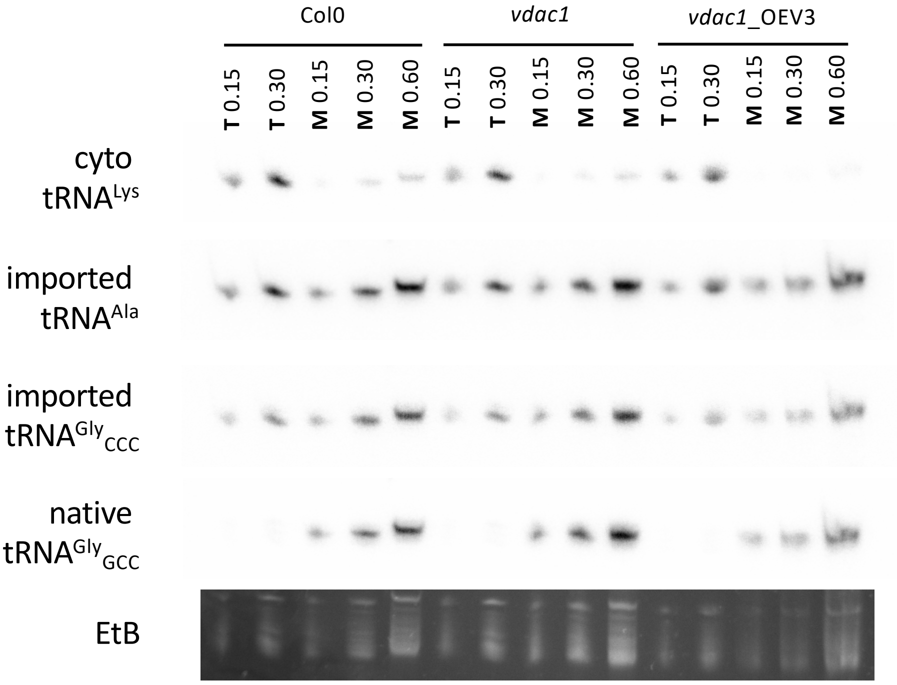

Membrane NB03:

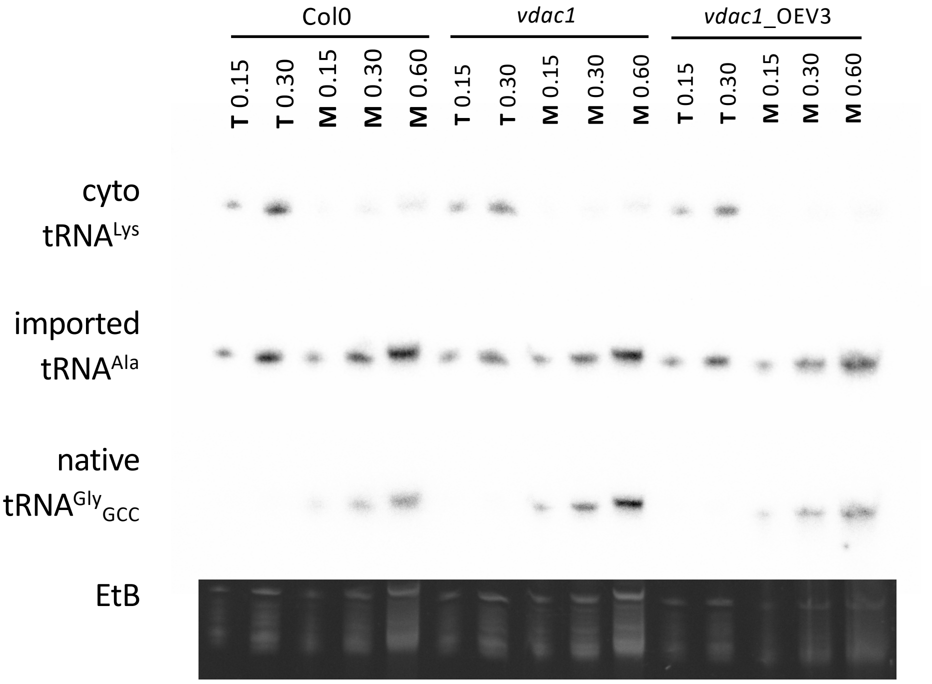

Raw data for Figure 2: quantification

NB	microg	lignée	extrait	mito Gly	imported Ala	cyto Lys	imported Gly
NB02	0.15	Col0	cyto	388496		4613787	2410440
NB02	0.3	Col0	cyto	942384	2766520	11981621	6630624
NB02	0.15	Col0	mito	7276139	1095295	342626	3280330
NB02	0.3	Col0	mito	17402589	2569583	1126957	9481583
NB02	0.6	Col0	mito	30999370	4639728	2432013	16143623
NB02	0.15	*vdac1*	cyto	414787	1258027	4221895	2122389
NB02	0.3	*vdac1*	cyto	884080	2410254	10617612	5942374
NB02	0.15	*vdac1*	mito	9533721	1160934	227315	3690952
NB02	0.3	*vdac1*	mito	21610449	2537812	568897	8903140
NB02	0.6	*vdac1*	mito	41177915	4909016	1522434	18132908
NB02	0.15	*vdac1* _OEV3	cyto	352179	959623	3243513	1703176
NB02	0.3	*vdac1* _OEV3	cyto	600893	2586915	9906286	5066745
NB02	0.15	*vdac1* _OEV3	mito	7504364	1448023	164884	2988675
NB02	0.3	*vdac1* _OEV3	mito	12724345	2096905	208011	4866984
NB02	0.6	*vdac1* _OEV3	mito	35478324	5756270	716973	15917821
NB03	0.15	Col0	cyto	232113	4914684	8494841	
NB03	0.3	Col0	cyto	915820	17835080	37137300	
NB03	0.15	Col0	mito	7309319	5191706	1072435	
NB03	0.3	Col0	mito	26384769	14933578	2682995	
NB03	0.6	Col0	mito	59373203	28586592	5876823	
NB03	0.15	*vdac1*	cyto	590746	5676120	10606466	
NB03	0.3	*vdac1*	cyto	918224	12332363	25581276	
NB03	0.15	*vdac1*	mito	14439183	5166538	802088	
NB03	0.3	*vdac1*	mito	45276172	12428440	1322948	
NB03	0.6	*vdac1*	mito	100804977	24869645	3508463	
NB03	0.15	*vdac1* _OEV3	cyto	471862	5628948	8996178	
NB03	0.3	*vdac1* _OEV3	cyto	636347	12291468	23904685	
NB03	0.15	*vdac1* _OEV3	mito	8334093	3936156	532571	
NB03	0.3	*vdac1* _OEV3	mito	31704297	12855667	901824	
NB03	0.6	*vdac1* _OEV3	mito	77112474	29036564	2224320	

Raw data for Figure 3: Ct

Stress	Nomenclature	
Control	T	
Cold stress	CS	4°C, 3h
Heat stress	HS	37°C, 3h
Oxidative stress	H2O2	H2O2 20mM, 24h
Salinity stress	SS	NaCl 150 mM, 24h
Phosphate starvation	P-	no Phosphate, 24h

Biological repeats:
1; 2; 3; 4; 5

primers

gene	Efficaciency	oligo	seq
RPL2	1.945	qPCR direct	CCGAAGACGGATCAAGGTAA
		qPCR reverse	CGCAATTCATCACCATTTTG
GAPDH	1.967	qPCR direct	aggctgctgctcacttgaa
(At1g13440)		qPCR reverse	aacatgggcgcatctttg
RPL12	1.887	qPCR direct	gacgtgtacgtccgagtaacc
		qPCR reverse	gaccgattttgggagctaga
VDAC3 long	2.008	qPCR direct	tccatatcttttacttggttctctctt
		qPCR reverse	gggaactccaaatggaacaa
VDAC3	1.974	qPCR direct	cactgaaatcggcaaaaagg
		qPCR reverse	tgttcccgttgtagtgatcg
VDAC1	1.984	qPCR direct	GCTCTTGTTCTTTCGATTCTCAG
		qPCR reverse	TGGTCACTGTTGTGGTCTTTG
VDAC2	1.974	qPCR direct	CCGATCTCTCTCAATCTCCG
		qPCR reverse	AGTCTCTCGTCAACAGATCTTTGG
VDAC4	1.914	qPCR direct	CGTCGATCTTCCATTTTCG
		qPCR reverse	AATCCTTGTTTAGGAGATCTTTGG

Salinity stress: mean Ct (mean of technical repeats)

Oligo	Dilution	SS1	T1	SS2	T2	SS3	T3	oligo	Efficiency E
RPL12	-1	20.713	20.653	22.295	22.285	21.045	21.730	RPL12	1.887
VDAC1	-1	19.040	18.817	22.385	21.670	18.885	19.175	VDAC1	1.984
VDAC2	-1	19.723	19.800	22.485	22.065	19.790	20.425	VDAC2	1.974
VDAC3	-1	17.810	17.987	20.870	20.625	17.795	17.985	VDAC3	1.974
VDAC3 long	-1	25.070	27.630	26.155	28.235	25.400	27.375	VDAC3 long	2.008
VDAC4	-1	22.587	22.440	25.750	25.010	22.515	22.870	VDAC4	1.914
RPL12	0	17.483	17.097	18.890	18.785	17.820	18.225	RPL12	1.887
VDAC1	0	16.130	15.860	19.290	18.515	15.890	16.210	VDAC1	1.984
VDAC2	0	16.633	16.667	19.145	18.785	16.645	17.035	VDAC2	1.974
VDAC3	0	14.710	14.787	17.555	17.250	14.585	14.780	VDAC3	1.974
VDAC3 long	0	22.770	25.003	23.800	25.350	23.585	24.910	VDAC3 long	2.008
VDAC4	0	19.237	19.070	22.155	21.640	19.160	19.370	VDAC4	1.914

Oxidatice stress: mean Ct (mean of technical repeats)

Oligo	Dilution	T1	H202-1	T2	H202-2	T3	H202-3	Oligo	Efficiency E
RPL12	-1	23.18	23.08	20.78	21.82	21.315	21.505	RPL12	1.887
VDAC1	-1	20.76	20.68	17.79	18.9	18.195	18.24	VDAC1	1.984
VDAC2	-1	21.15	21.22	18.52	19.725	18.92	18.945	VDAC2	1.974
VDAC3	-1	19.86	19.44	16.445	17.62	16.87	17.18	VDAC3	1.974
VDAC3 long	-1	29.28	27.53	27.845	27.25	27.935	27.935	VDAC3 long	2.008
VDAC4	-1	23.62	23.67	22.02	23.39	22.455	22.555	VDAC4	1.914
RPL12	0	19.70	19.76	17.305	18.24	17.905	18.085	RPL12	1.887
VDAC1	0	17.69	17.77	14.765	15.885	15.07	15.365	VDAC1	1.984
VDAC2	0	17.99	18.08	15.305	16.59	15.68	15.885	VDAC2	1.974
VDAC3	0	16.62	16.25	13.355	14.545	13.715	14.375	VDAC3	1.974
VDAC3 long	0	26.72	25.54	27.415	27.345	27.355	27.55	VDAC3 long	2.008
VDAC4	0	19.99	20.40	18.725	19.9	19.04	19.16	VDAC4	1.914

Heat stress: mean Ct (mean of technical repeats)

Oligo	Dilution	HS1	T1	HS2	T2	HS3	T3	oligo	Efficiency E
RPL12	-1		25.093	22.537	22.663	23.550	24.485	RPL12	1.887
VDAC1	-1		22.883	20.803	21.037	21.210	21.805	VDAC1	1.984
VDAC2	-1		23.903	21.423	21.687	21.705	22.330	VDAC2	1.974
VDAC3	-1		22.117	19.183	19.737	18.995	21.110	VDAC3	1.974
VDAC3 long	-1		29.700	26.743	27.600	27.605	30.400	VDAC3 long	2.008
VDAC4	-1		26.443	23.997	24.037	24.110	24.785	VDAC4	1.914
RPL12	0	21.183	20.873	19.300	19.323	20.090	20.625	RPL12	1.887
VDAC1	0	19.690	18.973	17.973	18.093	18.330	18.705	VDAC1	1.984
VDAC2	0	20.070	19.903	18.360	18.563	18.535	18.945	VDAC2	1.974
VDAC3	0	17.207	17.980	16.080	16.570	15.895	17.905	VDAC3	1.974
VDAC3 long	0	24.910	26.300	24.627	25.887	25.530	27.455	VDAC3 long	2.008
VDAC4	0	21.807	22.023	21.090	21.150	20.730	21.045	VDAC4	1.914

Cold stress: mean Ct (mean of technical repeats)

Oligo	Dilution	CS1	T1	CS2	T2	CS3	T3	CS4	T4	CS5	T5	oligo	Efficiency E
RPL12	-1	25.677	25.093	22.847	22.663	22.643	23.140	23.490	23.435	23.490	23.235	RPL12	1.887
VDAC1	-1	23.970	22.883	21.270	21.037	20.680	20.893					VDAC1	1.984
VDAC2	-1	24.283	23.903	21.827	21.687	20.957	21.427					VDAC2	1.974
VDAC3	-1	22.587	22.117	19.810	19.737	19.783	20.113	20.035	20.820	20.620	20.480	VDAC3	1.974
VDAC3 long	-1			25.113	27.600	26.153	29.753	27.985	30.730	27.110	29.100	VDAC3 long	2.008
VDAC4	-1	26.213	26.443	23.930	24.037	22.833	23.640					VDAC4	1.914
RPL12	0	20.830	20.873	19.543	19.323	19.413	19.807	20.145	20.360	20.240	20.155	RPL12	1.887
VDAC1	0	19.327	18.973	18.340	18.093	17.767	17.817					VDAC1	1.984
VDAC2	0	19.680	19.903	18.670	18.563	17.847	18.107					VDAC2	1.974
VDAC3	0	18.060	17.980	16.657	16.570	16.693	16.880		17.740	17.480	17.395	VDAC3	1.974
VDAC3 long	0			22.910	25.887	24.027	27.047			25.620	28.080	VDAC3 long	2.008
VDAC4	0	22.217	22.023	20.903	21.150	19.553	20.070					VDAC4	1.914

Phosphate starvation: mean Ct (mean of technical repeats)

oligo	Dilution	P-1	T1	P-2	T2	P-3	T3	Oligo	E
GAPDH	-1	19.413	19.483	19.610	19.835	19.050	19.115	GAPDH	1.967
RPL12	-1	23.173	20.950	22.710	21.795	22.710	21.585	RPL12	1.887
RPL2	-1	21.473	21.043	21.265	21.180	20.475	21.035	RPL2	1.945
VDAC1	-1	20.887	18.807	20.935	19.625	20.455	19.100	VDAC1	1.984
VDAC2	-1	21.090	19.897	21.555	20.625	20.820	20.210	VDAC2	1.974
VDAC3	-1	19.857	17.953	20.020	18.695	19.280	18.155	VDAC3	1.974
VDAC3 long	-1	30.043	26.450	30.000	27.805	30.135	27.605	VDAC3 long	2.008
VDAC4	-1	23.603	22.573	23.700	23.275	23.615	22.660	VDAC4	1.914
GAPDH	0	16.123	16.297	16.620	16.700		15.940	GAPDH	1.967
RPL12	0	19.623	19.310	19.270	18.540	19.475	18.200	RPL12	1.887
RPL2	0	18.197	17.737	18.090	17.945	17.215	17.695	RPL2	1.945
VDAC1	0	17.763	15.793	17.910	16.670	17.475	16.080	VDAC1	1.984
VDAC2	0	17.820	16.717	18.510	17.520	17.720	17.065	VDAC2	1.974
VDAC3	0	16.613	14.763	16.905	15.570	16.200	14.985	VDAC3	1.974
VDAC3 long	0	27.737	24.827	27.490	27.015	28.475	25.615	VDAC3 long	2.008
VDAC4	0	20.113	19.050	20.360	19.915	20.400	19.185	VDAC4	1.914

Raw data for Figure 5: in nmol O2 / min /100 mg seedlings

Raw Data (in nmol O_2 / min /100 mg seedlings)				
day	#	line	Total	After KCN addition
d1	a	Col0	21.06	6.29
d1	a	*vdac1*	16.20	7.21
d1	a	*vdac1* _OEV3	15.59	5.55
d1	a	*vdac3*	17.03	5.40
d2	a	Col0	14.63	4.58
d2	b	Col0	11.57	5.91
d2	a	*vdac1* _OEV3	10.43	5.55
d2	a	*vdac1*	13.94	6.81
d2	b	*vdac1*	13.37	8.22
d2	a	*vdac3*	11.44	8.07
d3	a	Col0	17.05	6.15
d3	b	Col0	14.03	5.03
d3	a	*vdac1* _OEV3	10.99	4.97
d3	a	*vdac1*	12.68	6.08
d3	b	*vdac1*	10.26	7.64
d3	a	*vdac3*	11.56	5.63
